# Post-Epidemic Distribution of Schmallenberg Virus in *Culicoides* Arbovirus Vectors in Poland

**DOI:** 10.3390/v11050447

**Published:** 2019-05-16

**Authors:** Julia Kęsik-Maliszewska, Magdalena Larska, Áine B. Collins, Jerzy Rola

**Affiliations:** 1 Department of Virology, National Veterinary Institute, 24-100 Puławy, Poland; julia_kesik@wp.pl (J.K.-M.); jrola@piwet.pulawy.pl (J.R.); 2 Department of Agriculture Food and the Marine, C/o Centre for Veterinary Epidemiology and Risk Analysis, UCD School of Veterinary Medicine University College Dublin, Belfield, D04 W6F6 Dublin 4, Ireland; aine.collins@agriculture.gov.ie

**Keywords:** Schmallenberg virus, *Culicoides*, transovarial transmission, overwintering, male *Culicoides*

## Abstract

Pooled samples of female and male *Culicoides* midges (5146 and 332 pools, respectively) that corresponded to a total number of 124,957 specimens were collected between 2013–2017 in the vicinity of cattle barns that were distributed throughout Poland were analyzed for the presence of Schmallenberg virus (SBV) RNA. Sixty-six pools tested positive (1.2%) with mean C_t_ value of 34.95. The maximum likelihood estimated infection rate (MLE) was calculated at 0.53 per 1000 individuals; however, it peaked in 2016 with MLE of 3.7. Viral RNA was detected in *C. obsoletus/scoticus* complex, *C. punctatus*, and C. *pulicaris* pools. Moreover, viral material was present in nulliparous (virgin) *Culicoides* females (MLE 0.27) and for the first time reported in males (MLE 0.34), which suggests the possibility of transovarial route of SBV or virus RNA transmission, as both do not fed on host blood. The accuracy of targeted versus random SBV surveillance in *Culicoides* vectors was compared. The relationship between infection rate (expressed as minimum infection rate; MIR), in addition to MLE, was compared with the density of virus infected midges (DIM). In conclusion, the SBV infection rate in the vector was significantly higher in 2016 as compared to other surveillance years; this is consistent with the simultaneous increase in SBV seroprevalence (seroconversion) in ruminants during the same year.

## 1. Introduction

Schmallenberg virus (SBV) (*Peribunyaviridae* family, *Orthobunyavirus* genus) was first detected in Germany in 2011 [1]. Over the course of the following two years, SBV overwintered and rapidly spread across Europe, reaching within-herd and between-herd seroprevalence of up to 100% in many locations [1,2,3]. Currently (May 2019), SBV is endemic in Europe, with cyclical epidemics occurring every 4–5 years [4]. The impact of SBV infection on European livestock production systems remains to be fully determined. SBV infection is reported in a range of domestic ungulates, including bovids, cervids, suids, and also in wild ungulates, such as giraffes and elephants in European zoos. Clinical signs associated with SBV infection in adult animals are typically mild and non-specific, such as pyrexia, diarrhoea, and drop in milk yield. However, if vertical SBV transmission occurs in pregnant females during the gestation susceptible period, then it may result in abortions, stillbirths, and congenital malformations. Horizontal SBV transmission (via direct contact, fomites etc.) between animals has not been confirmed [5]. The possibility of transovarial SBV (or other orthobunyaviruses) transmission in *Culicoides* has not (yet) been experimentally proven [6,7], however surveillance studies suggest that SBV, or at least its RNA, may descend into subsequent generations of *Culicoides* [8]. To date (May 2019), a number of studies have confirmed SBV infection in *C. obsoletus*, *C scoticus*, *C. dewulfi*, *C. chiopterus*, *C. pulicaris*, *C. punctatus*, and *C. imicola*, and thus implicate these species as possible competent vectors of the virus in Europe [5,6,7,8,9,10,11]. *Culicoides* Latreille (Diptera: Ceratopogonidae) biting midges become infected following the ingestion of a blood meal from a viraemic animal (in domestic ruminants SBV viraemia typically lasts 5–7 days, while seroconversion typically occurs 10–14 days following initial SBV infection) [5]. Virus transmission from the vector to host occurs when infectious female *Culicoides* bite immunologically naïve animals and transmit the virus to the host via their saliva. Only adult female *Culicoides* take blood meals (several times in their lifespan, approximately every 3–5 days), which are required for reproduction and egg maturation [12]. The mechanism of overwintering of arboviruses, such as SBV and bluetongue virus (BTV), is not yet fully understood. However, possible hypotheses have been proposed, including virus persistence in utero in infected foetuses, in wildlife reservoirs, or virus persistence in *Culicoides* vectors via transovarial transmission during which larvae and pupae may survive the winter in protected breeding places [8,13].

Moreover, *Culicoides* are known arbovectors of other emerging and re-emerging viruses of veterinary importance, such as Akabane virus (AKAV; *Peribunyaviridae* family, *Orthobunyavirus* genus) and vBTV (*Reoviridae* family, *Orbivirus* genus) and African horse sickness virus (AHSV; *Reoviridae* family, *Orbivirus* genus). The unprecedented emergence of novel viruses, such as BTV and SBV, in Europe is thought to be connected to changes that were observed in the modern world, such as globalization, climate change, intensification of trade, and intercontinental travel. Global warming may also favour the spread/expansion of afrotropical *Culicoides* species, such as *C. imicola*, beyond their existing geographical areas, which in turn may facilitate indigenous *Culicoides* species to gain the vector capacity of novel pathogens [14].

The aim of the present study was to evaluate the presence of SBV in *Culicoides* populations in Poland in the years following the initial Polish SBV epidemic that occurred in 2012. Bearing in mind previous studies that suggested possible transovarial SBV transmission in *Culicoides* [8], this study also aimed to explore the possibility of transovarial transmission in *Culicoides* by testing all parity forms of female (nulliparous, blood fed, parous, and gravid) specimens and also male specimens for SBV. The relationship between minimum infection rate (MIR), in addition to Maximum likelihood estimated infection rate (MLE,) was compared with to the density of virus infected midges (DIM) in order to assess the accuracy of calculated infection rates indicators. Targeted versus random surveillance in *Culicoides* sampling were discussed.

## 2. Materials and Methods

**Figure 1:**
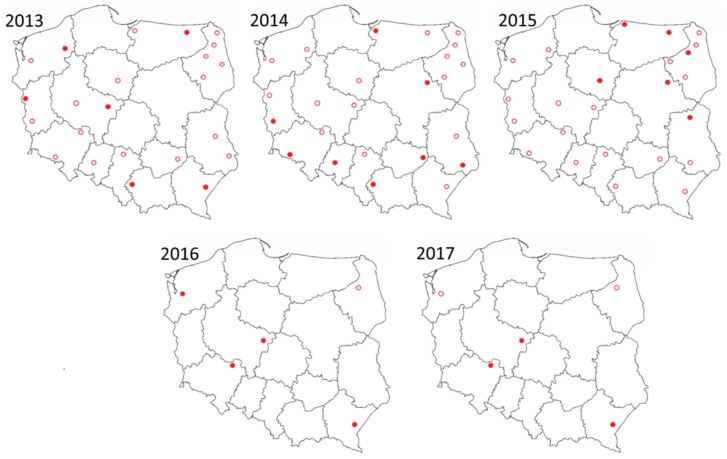
*Culicoides* trapping locations in Poland between 2013 and 2017. Red circle represents location of trap were no positive samples were detected, while red disc trap locations indicate where SBV was detected in *Culicoides*. From 2016 only targeted surveillance in five locations was performed.

The *Culicoides* were trapped and then tested for SBV in 23–24 locations in Poland from April to November 2013–2015. Following the guidelines of Gu et al. [15], targeted surveillance was conducted in five locations from April to October in 2016 and 2017 (Figure 1). Collection site locations were selected based on the abundance of vectors, evidence (confirmed PCR positives) of previous SBV circulation in *Culicoides*, and a history of recent seroconversion in domestic ruminants, according to the national SBV surveillance program [8,16]. Insect trapping commenced before the expected vector active period (based on data from previous years) and ended when three consecutive taps were found to be empty. UV-black light Onderstepoort-type (OVI) traps were placed in the proximity of cattle (up to 100 m) outside the barn and operated from dusk until dawn at weekly intervals.

The insects were placed in 70% ethanol pending morphological examination to species level using the taxonomic key of Glukhova [17] (except for *Culicoides obsoletus* and *Culicoides scoticus* females, which can only be distinguished on a molecular basis, therefore were combined into *C. obsoletus/scoticus* complex), gender, and parity status (nulliparous—unpigmented abdomen; parous—pigmented; gravid—abdomen filled with egg batches; freshly blood fed) under stereomicroscope (Olympus SZX16) based on previous study [18]. When possible, the pools consisted of approx. 23 female *Culicoides* for each species and physiological status. In total, 5146 pooled samples of female *Culicoides* were analysed. Male specimens were less abundant; therefore, the pools were created with specimens from one species and a single night catch collection resulting in 332 pools (average 17.7 individuals per pool). Weather data (minimum and maximum temperatures, wind speed, and rain fall) during the night catch collections was also recorded.

Using Lysing Matrix D Tubes (MP Biomedicals, Illkrich, France) with 1.4 mm ceramic beads, the insect pools were homogenized in RLT Buffer (Qiagen, Hilden, Germany). RNeasy Mini Kit (Qiagen) with QIAcube automated station (Qiagen, Hilden, Germany) was used to extract total RNA. Duplex real-time RT-PCR was performed for the simultaneous detection of SBV S segment [1] and culicoid 18S rRNA [8]. Primers and TaqMan probe for SBV amplification were used, as described by Hoffmann et al. [1], while the oligonucleotides for 18S used in a previous study [8] were slightly modified by adding the second TaqMqan probe (Cobs-18S-278: 5’Hex-ACTTTGGAGTGAGTACAAATGTACA3’-BHQ1). The in-house optimized real-time reverse transcriptase-polymerase chain reaction (QRT-PCR) using AgPath-ID One-Step RT-PCR Reagents (Ambion, Applied Biosystems, Courtaboeuf, France) kit in StepOne Real-Time PCR system (Applied Technologies, Foster City, CA, USA) was performed.

The sensitivity and efficiency of the extraction and amplification method were assessed using DNA standard prepared, as described previously [19]; as follows: RNA from SBV positive brain homogenate was extracted and amplified using the primers flanking SBV-S fragment [1] and Super Script III One-Step RT-PCR System (Invitrogen, Carlsbad, CA, USA). The product was electrophoretically separated in 3% agarose gel stained with ethidium bromide. The specific amplicon (SBV-S) of 87 bp was cut out from the gel and then purified using QIAguick Gel Ekstraction Kit (Qiagen). Nucleoid acid concentration was measured with Nanophotomether P330 (Implen) and the number of SBV-S copies was calculated using Avogadro constant. Tenfold dilutions of standard in SBV negative *Culicoides* midge homogenate that were pooled from several samples in RLT were prepared and then tested in triplicate by real-time RT-PCR, as described above. The efficiency of this process was computed at 85.4%. The lower efficiency was probably due to some inhibiting factors being present in *Culicoides* homogenates. C_t_ values of the tenfold dilutions of the SBV S segment fragment standard were used to draw a regression curve for sample viral load estimation, as presented in Figure S1.

A positive (C_t_ approx. 30, estimated copy number 10^3.9^), negative, and no template (for QRT-PCR) controls were included in each run. The PCR reaction was repeated for 42 cycles. Each SBV positive sample was repeated in duplicate. The results were calculated as the mean C_t_ (threshold cycle) value. The real-time RT-PCR cut of value (C_t_) below 40.5 (estimated copy number 10^1.2^) was considered to be positive. The implemented cut of value was slightly elevated as compared to a previous study [8]; this was chosen in order to evaluate vertical infection SBV transmission in nulliparous (virgin) female and male midges *Culicoides*. Therefore, every trace, repeatable signal was considered as positive.

### Statistics

The infection rate (IR) per 1000 individuals was calculated by the maximum likelihood estimation (MLE) using PoolInfRate version 4.0 software (Canters for Disease Control and Prevention, Atlanta, GA, USA) developed for the entomological studies (www.cdc.gov/ncidod/dvbid/westnile/software.htm). The minimum infection rate (MIR) is defined as number of positive pools, divided by number of tested individuals, multiplied by 1000. MLE and MIR were calculated in respect to all of the midge species tested (also for unconfirmed SBV vectors), from all locations (also for locations were no positive samples were found), except situations directly described in the text.

Midge mean abundance per single catch in respective species, parity status/gender was calculated as the sum of trapped individuals from all sites and years of study divided by the total number of night catches. Temporal mean abundance per single catch was computed as the number of collected midges in the respective timespan, divided by the number of night catches that were conducted during that period. Subsequently, the indicator of risk exposure: DIM (the density of infected midges), was calculated as the product of the mean midge abundance per single catch, multiplied by maximum likelihood estimation of infected individuals (MLE), divided by 1000.

SBV prevalence in the midges was compared in respect to different parameters (species, parity status/gender, year, and place of collection) using χ^2^ test and logistic regression. Since the distribution of C_t_ values was not normal (Shapiro–Wilk’s W = 0.9; *p* < 0.00001), Kruscal–Wallis or Mann–Whitney tests were implemented to study the differences in C_t_ values between different categories. Mean, minimum, maximum, and 95% confidence interval (CI) values were used in the descriptive statistics. Kurtosis was also calculated for Ct values. Statistical analysis was performed using STATA software version 13.1 (StataCorp, College Station, TX, USA)

## 3. Results

Over three million and four hundred thousand midges were trapped in over two thousand catches in all of the sampling years and locations. The most abundant species identified was the *C. obsoletus/scoticus* complex accounting for 62.4% (average 1289 individuals per single catch; ind./catch) of all *Culicoides* identified. *C. punctatus* accounted for 27.9% (average 577 ind./catch,), while *C. pulicaris* accounted for 15% (average 20 ind./catch.). Approximately 179 other *Culicoides* species were identified in each catch, none of which tested positive for SBV. Nulliparous females were the most abundant (60%; average 809 ind./catch) of all the *Culicoides* identified, followed by parous (23%; average 315 ind./catch) and blood fed (14%; average 188 ind./catch). Gravid females and males accounted for approx. 1% (average 12 ind./catch) of all *Culicoides* identified. The data mentioned above were variable for each trap and even consecutive catches from the same location could significantly differ. Detailed information regarding species and parity status/gender composition are presented in Table S1 and used for the calculation of mean *Culicoides* abundance in each individual catch in respective group of interest.

Out of 5478 prepared pools, 66 (1.2%; 95%CI: 0.9–1.5%) tested positive for SBV S segment with mean C_t_ 34.95 (range 22.1–40.3; kurtosis: 3.04; estimated copy number 10^2^.). The overall MLE was 0.53 (95% CI: 0.41–0.67). The internal control of culicoid 18S was simultaneously detected in QRT-PCR in most pools (86.4%), with the mean C_t_ value of 21.5 (range: 11.1–39.6; kurtosis: 3.0). In the remaining 745 samples in which the 18S control was not detected by QRT-PCR, 3% agarose gel electrophoresis confirmed the presence of the specific PCR product.

Between 2013 and 2017, SBV was detected in 19 sites from 13 provinces (Figure 1). The highest abundance of SBV positive midge pools (3.6%) was observed in samples that originated from Pikulice in podkarpackie province (49°44′52″ N; 22°47′37″ E). The second highest was Ewinów (2.5%) in wielkopolskie province (51°58′59″ N; 18°42′26″ E) and the third highest was Lelików (1.2%) from dolnośląskie province (51°33′31″ N; 17°25′25″ E). In these sites, positive samples were detected in three out of five sampling seasons.

**Figure 2:**
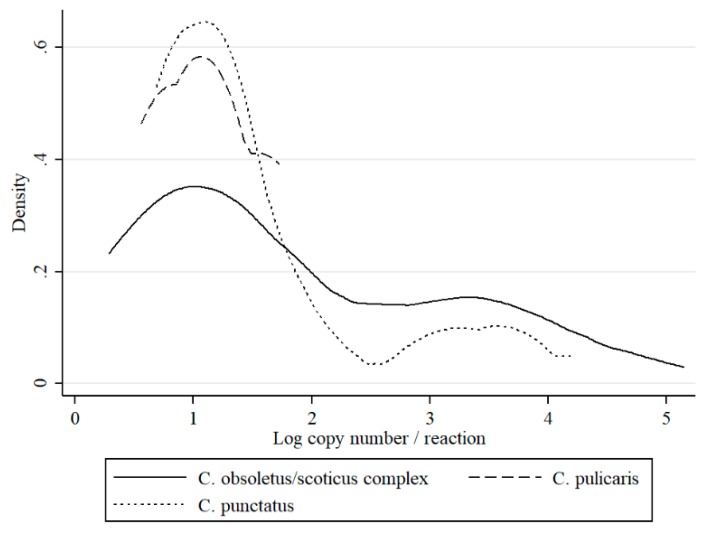
Distribution of estimated RNA copy numbers per sample (logarithm) values of optimized real-time reverse transcriptase-polymerase chain reaction (QRT-PCR) for Schmallenberg virus (SBV) detection by kernel density plot in respect to *Culicoides* species.

Pools were prepared from the most abundant midges belonging to 17 species (Table 1). Where the number of species was insufficient to create a pool, the specimens were not tested. Schmallenberg virus RNA was detected in four *Culicoides* species: *C. obsoletus/scoticus* complex, (43/3077 positive pools; 1.4%), *C. punctatus* (19 /2183 positive pools; 0.9%), and *C. pulicaris* (4/99 positive pools; 4.0%, all collected in the year 2016). For *C. pulicaris*, the MLE was the highest but DIM the lowest (Table 1). *C. obsoletus/scoticus* complex pools represented mean MLE, but the highest DIM. For *C punctatus* calculated MLE was the lowest and DIM comparable with *C. pulicaris*. When the differences in C_t_ values between *Culicoides* species was considered, the highest loads of virus (which corresponded to the lowest mean C_t_ value) were observed for the *C. obsoletus/scoticus* complex (34.4; estimated copy number 10^2.8^; kurtosis: 2.5) and *C. punctatus* (35.7; estimated copy number 10^2^; kurtosis: 4.4), while the mean C_t_ value for *C. pulicaris* pools (37.3; estimated copy number 10^2^; kurtosis: 1.3) was slightly higher; however, these differences were not statistically significant. Two peaks around 10^1^ and 10^3.5^ at the distribution curves of RNA copy number per sample were observed for the *C. obsoletus/scoticus* complex and *C. punctatus* (Figure 2).

**Figure 3:**
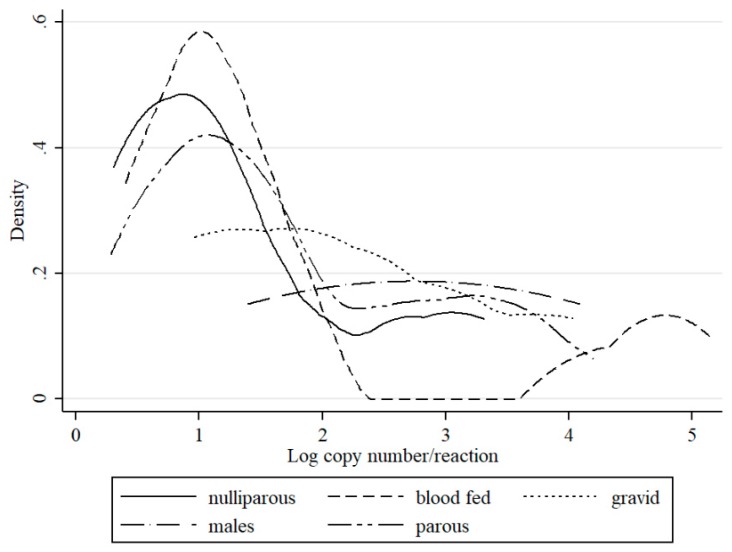
Distribution of estimated RNA copy numbers per sample (logarithm) values of QRT-PCR for SBV detection by kernel density plot with respect to gender or female parity status.

Schmallenberg virus RNA was detected in midge pools that represent each parity status. SBV detection was higher in in gravid *Culicoides* (2.9%; MLE 1.43) as compared to parous females (1.9%; MLE 0.84), (*p* < 0.05). Blood fed *Culicoides* were positive in 1.1% (MLE 0.5) of pools. Notably, SBV RNA was also detected in nulliparous (0.6%; MLE 0.27) and male (0.6%; MLE 0.34) pools (Table 1). However, when the density with respect to gender and parity status composition of *Culicoides* in Poland was considered, the number of SBV positive parous females (DIM 0.26 positive individuals per 1000 trapped) was higher than nulliparous (DIM 0.22). Positive pools of blood fed females, gravid females as well as males *Culicoides* were significantly less common when compared to parous and nulliparous (DIM 0.09, 0.02, and 0.01, respectively). Bimodal distribution of detected SBV RNA loads was apparent in blood fed and parous females (Figure 3). In nulliparous specimens, two peaks could also be distinguished; however, the virus yields were much lower. In gravid female samples, the distribution of positive pools increased with decreasing RNA copy number in the pool. SBV was also identified in two male *Culicoides obsoletus* pools; remarkably, one of these pools comprising of only two specimens gave a C_t_ value for SBV of 26.0 (estimated copy number 10^5^).

**Figure 4:**
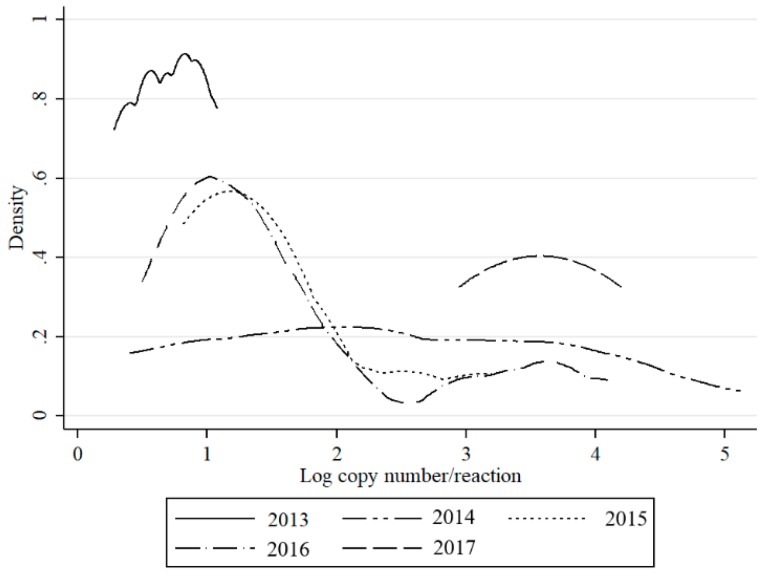
Distribution of estimated RNA copy numbers per sample (logarithm) values of QRT-PCR for SBV detection by kernel density plot in respect to year of sampling.

SBV genetic material in *Culicoides* vectors (*C. obsoletus/scoticus* complex, *C. punctatus*, *C. pulicaris*) was detected in approx. 0.5% samples in 2013, 2015, and 2017; and, 1% in 2014. In 2016, SBV peaked in *Culicoides* with 7.2% of pools testing positive (Table 1). The density of the infected midges (DIM) and MLE was also the highest in 2016 (5.85 and 3.71, respectively), while they were below 1 in other years. Bimodal distribution of detected RNA copy number values was observed in 2015 and 2016, while in 2014, the virus RNA yields were evenly distributed (Figure 4). In 2017, in contrast to 2013, higher virus loads were recorded (Figure 4).

In 2016 and 2017, only five trapping sites were sampled for the study. Site selection was based on the availability of regular and abundant catches, even geographical distribution (three different NUTS1: PL3, PL4, and PL5) and/or evidence of recurrent SBV circulation in *Culicoides*. In Table 2, the comparison of two surveillance approaches are presented; random surveillance that was conducted in 2013–2015 and targeted between 2013 and 2017, are presented. The results were comparable, except for 2014, where the MLE in targeted surveillance was over four times lower than that computed for random surveillance in 24 trap locations.

**Figure 5:**
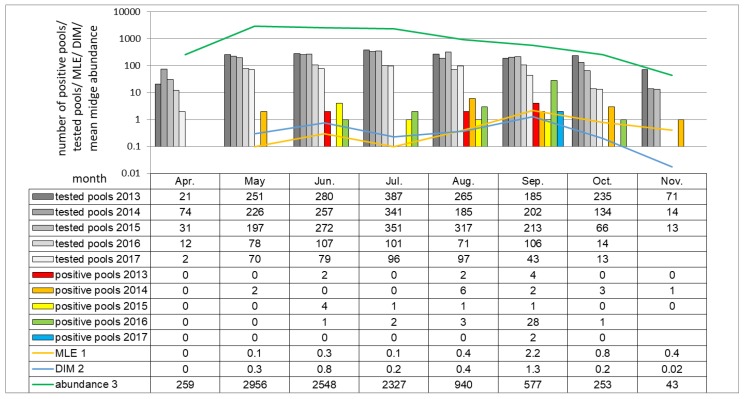
Monthly distribution of SBV RNA positive pools in all tested seasons (no pools were tested in November in 2016 and 2017). The shades of grey and colored columns represent the numbers of tested pools and SBV positive midge pools in respective year. The green curve represents the average number of individual midges caught in month intervals between 2013 and 2017 indicating the dynamic of midge month abundance over the course of the study. Similarly, the MLE and virus infected midges (DIM) dynamics are presented as yellow and blue curves, respectively. The original numeric data are placed below in the table. ^1^ MLE (Maximum likelihood estimation) calculated for 2013–2017; ^2^ DIM (Density of infected midges) calculated as proportion of mean abundance multiplied by MLE per 1000 individuals; ^3^ Abundance (Mean midge abundance per single catch) sum of midges trapped in consecutive month in 2013–2017 divided by number of catches in respective month.

When considering monthly schedule in all seasons, midge activity peak starts in May and last till July. The earliest SBV detection was made in May (2/822 pools; 0.24%), the latest in November (1/94 pools; 1.0%), in both cases in 2014 (Figure 5). SBV positive *Culicoides* pools were the most frequently detected in September (37/794 pools; 4.9%) than in August (12/935 pools; 1.3%). MLE, as well as DIM, were the highest in September (2.2 and 1.3 positive midge per 1000 trapped, respectively), than in June (0.3 and 0.8, respectively).

**Figure 6:**
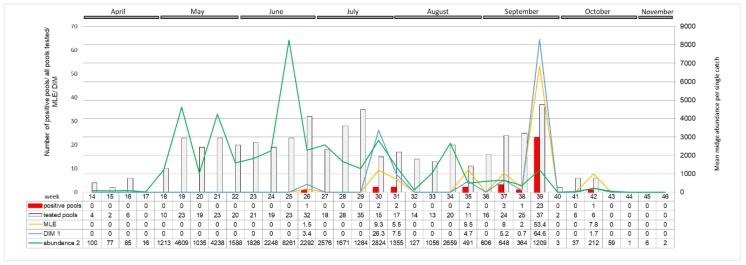
Weekly distribution of SBV RNA positive pools in 2016 (tested from April to October). The grey and red columns represent the numbers of tested and SBV positive midge pools. Green curve connecting the average number of individual midges caught in weekly intervals during 2016, corresponding to the right X-axis, represents the dynamics of midge week abundance. Similarly, the MLE and DIM dynamics are presented as yellow and blue curves, respectively. The original numeric data are placed below in the table. ^1^ DIM (Density of infected midges) calculated as proportion of mean abundance multiplied by MLE per 1000 individuals; ^2^ Abundance (Mean midge abundance per single catch) sum of midges trapped in consecutive week of 2016 divided by number of catches in respective week.

A distinct increase in the SBV positive pools of *Culicoides* with high virus RNA load was detected in autumn 2016. Positive midge pools were detected in three out of five locations sampled: in Pikulice (10 out of 11 pools positive), Lelików (five out of eight pools positive) and Ewinów (seven out of nine pools positive), between 28–30 September 2016 (week 39) (Figure 6). Schmallenberg virus RNA was detected in female *C. obsoletus/scoticus* complex, *C. punctatus*, and *C. pulicaris* in all parous statuses as well as in one male pool comprising 22 *C. obsoletus* specimens (*C*_t_ 36.1; estimated copy number 10^2.3^). The peak in SBV positive pools coincides with a small peak in *Culicoides* abundance per trap (approx. 1200 midges per trap). Computed MLE for all of the sampled locations reached 53.4 in week 39. DIM, respecting vector temporal midge abundance, in week 39 was approx. 65 infected *Culicoides* per 1000 trapped. The second peak in DIM (approx. 26 infected *Culicoides* per 1000) was noted in week 30 (late July), when MLE was only 9.3.

## 4. Discussion

This is the first study to describe the long-term SBV surveillance in *Culicoides* biting midges in five consecutive years. The results of this study indicate that monitoring SBV circulation in *Culicoides* is a suitable method to monitor post-epizootic SBV infection dynamics despite the fact that *Culicoides* trapping and preparation for SBV analysis is quite laborious and expensive. In the present study, an increase in SBV prevalence in *Culicoides* was evident, particularly during 2016. These findings are consistent with reports of SBV re-emergence and recirculation in ruminants in Poland (publication in preparation) and in other European countries [4,20,21,22,23,24]. These findings provide a satisfactory argument to consider that vector surveillance is a useful tool to monitor the SBV enzootic situation. However, there are also some limitations of studies that are based solely on QRT-PCR, such as inability to distinguish between non-infectious virus particles (viral RNA detected from insect digestive track, not disseminated infection) and virus multiplying in the insect salivary glands (disseminated infection, infectious virus). However, as previously discussed [8,23], C_t_ values may be used to predict the status of insect infection. Veronesi et al. [25] reported that C_t_ value below 24 in *Culicoides* most probably indicates transmissible infection in experimentally infected *Culicoides sonorensis*, while C_t_ above 32 suggested sub-transmissible infection. These estimations were determined for single insects or single head/abdomen specimens of midges that were infected in laboratory conditions. Therefore, such estimations should be adapted with caution to the field samples. In the present study, all of the groups except nulliparous had pools with C_t_ under 26. Distributions of C_t_ values (expressed as RNA copy number in Figure 2, Figure 3 and Figure 4) in the present study was bimodal for *C obsoletus/scoticus* complex and *C. punctatus*, but was shifted to the right when compared to Veronesi et al. [25] and previous work [8], (Figure 2). Two peaks can be observed corresponding to transmissible-disseminated infection around C_t_ = 28 and subtransmissible infection around C_t_ = 38. This shift to the right may be the result of the different extraction method being used (manual TRI versus automated RNeasy Mini Kit (Qiagen)) or, alternatively, by actual lower virus loads in the vector after first epidemic wave in 2011/2012 [8,21].

One limitation of studies that are based on *Culicoides* surveillance is the selection of tools that are available to estimate the true prevalence of infection. MIR or MLE can be used as infection rates for insects tested in pools for assessing the risk of arbovirus transmission. However, the reports suggest that it can be common to underestimate the prevalence of arbovirus infection in a mosquito population due to biased sampling, pooling and virus testing [26]. In the presented study, MIR and MLE for different midge groups were convergent (Table 1). The MIR calculations assume that, that in pooled samples, only one individual is positive, while MLE directly estimates the proportion of infected insects in the sample [15]. The discrepancy between those indicators is greater when the levels of insect infection are high leading to the underestimation of MIR. Those indicators should be used in conjunction with other indicators, such as vector population size, age structure, and weather- modulating extrinsic incubation period (EIP), as well as historical baseline data, when assessing the risk of arbovirus transmission. A comparison of MLE and DIM can lead to contradictory conclusions. For example, MLE was the highest for gravid females (1.43), while DIM shows that positive individuals were only trapped occasionally (0.02 positive midges per 1000 collected); this may be due to the low *Culicoides* abundance in this group. In comparison, for nulliparous, MLE scored only 0.27, but this group comprised 60% of trapped midges, thus the calculated DIM was relatively high (0.22 positive midges per 1000 trapped). Analysis of MLE and DIM in 2016 showed that, when MLE had comparable values (8–9.5), the true number of infected vectors differed. It was much higher in late July (week 30; 26.2 infected midges per 1000 trapped) than in late August (week 35; 4.7) or mid-September (week 37; 5.2). These differences are likely the result of higher mean midge abundance in 2016 from May to July, as compared to mid-August. However, it is important to note that midge abundance in each site and night catch collection can differ by up to two logs (Figure 3) [18]. Parous females that would be capable of transmitting SBV to naive animals; while a positive SBV PCR suggests that they are infected, they are not necessarily infectious (Table S1). Parous midges accounted for approximately 23% of all *Culicoides* that were identified. This means that, even when DIM reached its peak in week 39 in 2016 (64.6 infected midges per 1000 trapped), it is estimated that only 15 SBV positive parous females were trapped per 1000 midges. Microclimatic conditions for the virus transmission were favourable during the re-emergence and recirculation of SBV in 2016 in the sites where positive *Culicoides* were found. Recorded minimum and maximum temperatures during night trap collections were in the range of 11–13 °C and 16–23 °C, respectively, while no rain and only light winds) were recorded. One study reported that the threshold temperature for SBV replication is 12.3 °C [27]. Bearing this in mind, microclimatic temperatures in places where insects rest are generally higher than the standard meteorological temperature that was measured above the ground [28]. The results of the present study indicate that there was a possibility of SBV replication in vectors in late September 2016. The true infection risk (DIM) was elevated during summer and autumn 2016, this, in addition to favourable weather conditions, likely favoured virus transmission from *Culicoides* vectors to susceptible animal hosts. Estimated infection rate (MLE), as well as DIM in *Culicoides* vectors, reached their peaks in 2016 (5.85 infected midges per 1000 trapped, weakly up to 64.5); this corresponded with reports of SBV re-emergence and recirculation in Poland (data unpublished) and in other European countries [4,20,21,22,23]. Additionally, SBV seroconversion was also observed in wildlife in 2016 in Poland [24]. Akabane virus, which is a close relative to SBV, transmitted by several *Culicoides* species in Australasia, tends to occur every couple of years in a cyclical pattern. Akabane outbreaks are reported to occur when vectors invade new territories due to favourable climatic conditions or when population immunity is reduced, due to antibody decay, increased number imports of naive animals, or the birth of new generation of naïve animals [29]. It is likely that the documented reduction in SBV herd immunity in Europe facilitated the re-emergence and recirculation of SBV in 2016 [4,20,21,22,23,24].

The design of a baseline MIR above which SBV outbreak in the vector and the host may be suspected may be helpful in assessing an increased risk of SBV transmission. The MIR, however inadequate, as discussed above, was chosen due to the simplicity of method of calculation, requiring the least number of variables. In other European countries, MIR for SBV in midges was variable in different studies (Table 3). Those differences may be the result of spatiotemporal differences in true IR, abundance, and interactions of vector and host, as well as differences in midge sampling (different types of traps/trapping schedule), pooling method (different pool size, head/ whole insects, testing all trapped species/only confirmed SBV vectors, testing all parity status/only pigmented females), and in SBV RNA testing procedure itself. In most reports, MIR is calculated over 1.0 during the SBV emergence in 2011–2012, with the highest in Poland, Belgium, and the Netherlands: 5.3, 5.2, and 2.3, respectively [8,10,30]. During the SBV re-emergence in 2016, MIR was calculated at 7.1 in Belgium [20] and at 3.7 in Poland, as observed in the present study. Between those two infection waves, the first started in 2011–2012 and the second one started in 2016, SBV in *Culicoides* was only reported in Poland with MIR calculated at approx. 0.2–0.4. Therefore, these findings confirm the validity of MIR baseline for SBV infection in *Culicoides* vector at 1.0. The values under 1.0 should be considered as low infection risk for the host, while values of 1.0 and over equate to an elevated risk of transmission. The proposed baseline MIR should be computed for a sampling period at least every couple of months due to wide variations in the infection rate between catches (Figure 6), [20] and the number of individuals/pools.

As reported in this paper, total IR (expressed as MIR) as well as IR for particular species was consistent with previous papers describing the post epidemic situation, as demonstrated by Balenghien et al. [7] (Table 3). Based on DIM *C. obsoletus/scoticus* complex (0.79 positive individuals per 1000 trapped) are the dominant SBV vector species in Poland, followed by and *C. punctatus* (0.06). To date, SBV RNA has not been detected in *C. pulicaris* at high levels and, where it has been detected, the MIR values were high, ranging between 1–3.7 [8,9,32,33]. This may be due to the fact that MIR values are vulnerable to small sample sizes. The calculated DIM (0.04) as well as C_t_ over 34.9 (Figure 2) for *C. pulicaris* in the present study suggested non-disseminated infection [25], and therefore indicated a limited role of this species as a SBV vector. In our study, all of the positive *C. pulicaris* originated from a single site, from catches during SBV suspected outbreak in autumn 2016. Speculation may be made that *C. pulicaris* became infected during a wide distribution of SBV in *Culicoides* populations.

Aside from the aforementioned four species, which are the most common in Poland and found SBV positive, the remaining 13 species were insufficiently abundant to prepare numerous pools (from one to 37 pools from each species, Table 1). Therefore, the vector capacity of these species could not be determined in the present study; further research is recommended on a larger sample size.

The results were comparable despite that almost three times less midge pools were included in targeted (2013–2017) versus random surveillance (2013–2015), which confirmed that the sample size of targeted surveillance does not affect the assessment of SBV epizootic situation by vector monitoring (Table 2). Additionally, increased MLE observed by targeted surveillance in the country in 2016 corresponded to the situation in other reports [20]. The differences in MLE in 2014, as computed trough the targeted and random surveillance schemes, were not considered to be important, particularly if we would adapt MLE cut-off of one for the differentiation of low and high risk of SBV infection of the host. This makes the usefulness of targeted surveillance sampling approach (less labour and cost consuming) worth considering [15]. Additionally, the increased MLE observed by targeted surveillance in 2016 was consistent with an increase in SBV circulation in ruminants in a number of European countries [4,20,21,22,23,24]. Nevertheless, the identification of sites with adequate size catch collections and a greater likelihood of arboviral transmission to be determined require additional stages of a *Culicoides* surveillance program before targeted surveillance can be implemented. Vector monitoring should be carried regularly in as small as possible time intervals due to relatively short time when SBV (and other arboviruses) can be detected in *Culicoides* populations (Figure 6), as presented in this study and by other researchers [20]. That is related to a short and transmissible viraemia in the mammalian host and rapid spread of infection within a herd (a few infectious vectors can affect a large number of naïve animals within days). Finally, host population seroconvert, which inhibits the further dissemination of infection in the vector, and host population is important to consider [20,34,35].

Single positive individual species or parity status misclassification could have resulted in false positive results in pools from other species or parity statuses. However, such errors were likely limited when considering the highly qualified and experienced entomologist preparing pools for this study. Only specimens that were morphologically identified and confirmed were chosen to prepare the pools. Sexual dimorphism in *Culicoides* allows for clear differentiation between the sexes and limits the error.

In the present study, midges were trapped in the proximity of cattle barns. In those locations, the host-seeking midges, which are nulliparous and parous females, predominate, and are therefore more likely to be collected. This facilitates virus detection in the vectors, but also influences the evaluation of actual vector abundance and composition, thus affecting the calculation of DIMs for parity/sex and species level. As revised by Probst et al. [36], UV-light traps may underestimate the biting rates of some species (*C. chiopterus*, *C. scoticus*) and overestimate others (*C. imicola*). However, the presented trap composition reflects vector population in host surrounding as much as is achievable.

As discussed earlier [18], in respect to midge biology, only parous females representing half of identified positive samples, and 23% of trapped midges, can be considered to be active virus carriers. It is in this physiological status that insects feed on host species, sometimes multiple times on divergent species, which enables the live virus to be transmitted [37]. Hypothetical DIM for parous females was computed at 0.26 per 1000 individuals making this group the most affected. In this group, the detected RNA yields represented by the RNA copy number showed bimodal distribution, indicating, in at least some samples, that disseminated infection occurred (Figure 3). Therefore, our results confirm the function of parous females. However, the consistent detection of SBV positive pools comprising of midges that did not feed on animals, such as nulliparous (virgin) females as well as males, may be the indirect evidence of transovarial transmission of the virus within midge population, which has been previously suggested in studies [8]. Some research studies do not distinguish nulliparous females, but rather account for them as unpigmented or non-engorged group and testing together with parous and gravid females [10,31]. This approach may underestimate the actual number of infected midges in each group and makes it impossible to detect the number of potentially infectious parous females. In other studies, vertical SBV transmission is questioned due to the failed detection of positive nulliparous pools [5]. However, this may be also the result of different sample size, which is much larger when compared to a study that was conducted by de Regge et al. [5], who tested almost forty times less insects. In comparison, in the present study, only 14 SBV positive pools were detected out of 2218 nulliparous pools, and from those, only two had C_t_ around 29, which suggests active virus transmission [25]. However in general this group had relatively the highest mean C_t_ corresponding to the lowest virus RNA yield (Figure 3). In a previous study, the SBV C_t_ value in nulliparous females was also high, over 29, while C_t_ was generally lower than described in the present study in other groups [8]. Akabane virus was isolated from nulliparous females, while no virus was detected in laboratory reared from dung [6]. For bluetongue virus (BTV), an arbovirus that is also transmitted by *Culicoides*, the transovarial route of transmission in *Culicoides* has yet to be determined. In support of the theory of transovarial BTV transmission in *Culicoides*, BTV was detected in midge larvae, as well as in mature insects in the ovarian sheath, within the immature yolk bodies and vitelline membrane of the oocyte, as well as on eggs oviposited by BTV-infected females [38,39]. However, transovarial transmission could not be repeated in laboratory conditions [39]. Furthermore, Epizootic Haemorrhagic Disease virus (EHDV) was detected in midge ovarian sheath, but not in tissues that come into direct contact with the developing oocyte, which limits the possibility of infecting oocytes [40]. Vertical transmission was suggested for West Nile Virus (WNV) transmitted by *Culex*. Only females having high titre infections were capable of passing the virus to their progeny [41]. Transovarial transmission of Dengue virus (DENV) in *Aedes* spp. mosquitoes is important in virus persistence, therefore considered as part of xenomonitoring [42].

Another surprising observation, which suggests the possibility of vertical SBV transmission in midges, is the detection of viral RNA in the males. This is the first time that SBV RNA in male *Culicoides* is tested and positive pools are reported. Remarkably, the pool of two *C. obsoletus* male presented low C_t_ (26.0), and raises suspicion regarding disseminated infection within the vector [25]. Male midges do not feed on mammals, thus they can only become transovarialy infected or might have contracted the virus by contact with SBV infectious material (mechanical contamination). However, since the virus load detected in the present study would be akin to SBV infected brain tissue of new-born mammal or blood from a viraemic animal, this is quite unlikely to happen. Moreover, we can exclude laboratory contamination, as testing negative controls and retesting suspicious samples control the reaction. As mentioned above, the misclassification of sexes is unlikely in respect to clear sexual dimorphism in the midges. In some reports, the male insects were also suspected to pass other arboviruses vertically. In *Culex* females WNV [41], higher virus loads may increase the chances for such transmission. DENV was also detected in the field caught *Aedes* males, at comparable levels to SBV in the present study [43].

Considering engorged blood fed female *Culicoides* as SBV positive may be not be very prudent, as positive SBV RNA results may originate from the infected blood meal. Moreover, testing those pools can be considered as a part of xenosurveillance, in which insect vectors are treated as proxy for syringe sampling of the host. From this point of view, MLE in blood fed females may reflect infection rates in mammals. In studies on human pathogens, this approach corresponded with more traditional blood sampling methods [44]. Balengheim et al. [7] demonstrated that, following artificial inoculation of field caught *C. scoticus* with SBV, the virus RNA could be detected for at least eight days, with mean C_t_ indicative of virus replication within the vector, which could be longer than that of SBV viremia in mammals [5]. In the present study, calculated MLE in blood fed females was the highest in 2016 (3.5), as compared to 2014 (0.4). In other years, no positive engorged midge pools were detected. Further studies are recommended if the SBV infection rate in the vectors reflects infection rates in the host.

During female midge life cycle, blood intake is followed by oviposition, and it can occur several times. It results in blood fed and gravid groups being more exposed to SBV infection and superinfection. In gravid females, MLE was the highest (1.43) with respect to other parity groups; however, DIM remained low (0.02). In the blood fed, MIR was calculated to be 0.5, while DIM was 0.09 (Table 1). It is possible that the number of blood fed and gravid females may be underestimated, which in turn would have implications for the reliable calculation of DIM. Underrepresentation of blood fed and gravid females in catch collections may be explained by *Culicoides* biology; after the blood meal, they probably leave the host surrounding to produce and lay eggs, and they are less likely to be trapped outside the barns. In a previous study, high PCR replication rates (expressed as low C_t_) were noted in gravid and blood fed *Culicoides* in comparison to nulliparous *Culicoides*, but at a lower level when compared to parous *Culicoides* [8].

## 5. Conclusions

(1) SBV RNA can be detected in nulliparous *Culicoides* females, as well as in males, which suggests transovarial transmission of the virus or viral RNA. (2) *Culicoides obsoletus/scoticus* complex is the main SBV vector in Poland, while SBV RNA was detected in *Culicoides pulicaris* for the first time in Poland. (3) The infection rate of SBV within vector calculated as MLE or MIR may not be the most accurate and reliable indicator of risk exposure; midge spatio-temporal abundance should be considered and the density of infected midges (DIM) computed. (4) Baseline for MIR to assess the risk of SBV active circulation in the area using midge monitoring was suggested at 1.0 SBV positive *Culicoides* per 1000 individuals trapped. (5) A significant increase of SBV prevalence in the *Culicoides* in 2016 suggests the recirculation of the virus, as observed in other European countries. (6) The efficiency of targeted SBV surveillance in vectors was proven as a satisfactory tool to monitor the post-epizootic SBV situation in Poland.

From this study new questions arose: (1) Is transovarial transmission of SBV possible? (2) Does SBV amplify in nulliparous and male midges? Does nulliparous, after maturation, transmit the virus to mammals while feeding or male during mating to other female midges? (3) Do SBV titres in vectors reflect SBV titres in hosts, and can the blood fed female *Culicoides* be used as a xenosurveillance tool? (4) Can one rely on vector infection rates calculated as MIR or MLE without knowing actual insect abundance and DIM?

## Figures and Tables

**Table 1:** Sampling data, results of QRT-PCR for Schmallenberg virus (SBV) detection, and infection rate estimates.

Category	Positive Pools/Tested Pools	% of Positive Pools (95%CI ^1^)	Mean C_t_ (Min-Max)	Estimated Mean Log SBV Copy Number (Min-Max)	MIR ^2^	MLE ^3^ (95% CI)	DIM ^4^ (95% CI)
**Species**							
*C. achrayi*	0/5	0	-	-	0	0	0
*C. chiopterus*	0/37	0	-	-	0	0	0
*C. circumscriptus*	0/6	0	-	-	0	0	0
*C. deltus*	0/13	0	-	-	0	0	0
*C. fagineus*	0/3	0	-	-	0	0	0
*C. fascipennis*	0/1	0	-	-	0	0	0
*C. griscens*	0/1	0	-	-	0	0	0
*C. impunctatus*	0/13	0	-	-	0	0	0
*C. newsteadi*	0/8	0	-	-	0	0	0
*C. odibilis*	0/1	0					
*C. obsoletus/scoticus* complex	43/3077	1.4 (1.0–1.9)	34.4 (22.1–40.3)	10^3.7^ (10^0.3^–10^5.1^)	0.55	0.61(0.45–0.81)	0.79 (0.58–1.04)
*C. pulicaris*	4/99	4.0 (1.1–10.0)	37.3 (34.9–39.2)	10^3.0^ (10^0.7^–10^4.2^)	1.91	1.92 (0.63–4.59)	0.04 (0.01–0.09)
*C. punctatus*	19/2183	0.9 (0.5–1.4)	35.7 (25.6–38.8–37.5)	10^1.3^ (10^0.6^–10^1.7^)	0.38	0.39 (0.24–0.59)	0.06 (0.13–0.34)
*C. riethi*	0/29	0	-	-	0	0	0
*C. salinarius*	0/1	0	-	-	0	0	0
*C. tagineus*	0/1	0	-	-	0	0	0
**Parity Status of Female ^5^/Gender**							
nulliparous	14/2218	0.6 (0.3–1.1)	36.3 (28.9–40.2)	10^2.5^ (10^0.3^–10^3.3^)	0.27	0.27 (0.15–0.44)	0.22 (0.12–0.36)
parous	33/1734	1.9 (1.3–2.7)	34.9 (25.6–40.3)	10^3.0^ (10^0.3–^10^4.2^)	0.83	0.84 (0.59–1.16)	0.26 (0.19–0.37)
blood fed	11/978	1.1 (0.5–2.0)	35.0 (22.1–39.8)	10^4.2^ (10^0.4^–10^5.1^)	0.5	0.5 (0.26–0.87)	0.09 (0.05–0.16)
gravid	6/210	2.9 (1.1–6.1)	34.4 (26.2–37.8)	10^3.3^ (10^1.0^–10^4.0^)	1.41	1.43 (0.58–2.96)	0.02 (0.01–0.05)
male	2/332^6^	0.6 (0.07–2.2)	31.1 (26.0–36.1)	10^3.7^ (10^1.4^–10^4.0^)	0.34	0.34 (0.06–1.11)	0.01 (0.00–0.02)
**Year**							
2013	8/1695	0.5 (0.2–0.9)	38.7 (37.3–40.3)	10^0.7^ (10^0.3^–10^1.0^)	0.22	0.2 (0.09–0.38)	0.2 (0.09–0.38)
2014	14/1433	1.0 (0.5–1.6)	32.3 (24.8–39.8)	10^4.1^ (10^0.4^–10^5.1^)	0.41	0.4 (0.23–0.67)	0.73 (0.42–1.22)
2015	7/1460	0.5 (0.2–1.0)	35.9 (29.4–38.3)	10^2.4^ (10^0.8^–10^3.2^)	0.22	0.22 (0.1–0.43)	0.27 (0.12–0.53)
2016	35/489	7.2 (5.0–9.8)	35.3 (26.0–39.6)	10^3.0^ (10^0.5^–10^4.0^)	3.58	3.71 (2.63–5.09)	5.85 (4.14–8.02)
2017	2/400	0.3 (0.06–1.8)	28.0 (25.6–30.3)	10^3.9^ (10^3.0^–10^4.2^)	0.22	0.22 (0.04–0.73)	0.77 (0.14–2.57)

^1^ Confidence interval for binomial values (one-sided, 97.5% CI for 0%); ^2^ Minimum infection rate; ^3^ Maximum likelihood estimation of infection rate; ^4^ Density of infected midges from respective category calculated as proportion of mean midge abundance per single catch (sum of trapped midges divided by number of catches in respective group of interest) multiplied by MLE divided by 1000 individuals; ^5^ For 5 specimens parity status was not defined; ^6^ Males represented six species: *C. achrayi* (*n* = 1), *C. chiopterus* (*n* = 2); *C. obsoletus* (*n* = 182); *C. punctatus* (*n* = 136); *C. riethi* (*n* = 7); and *C. scoticus* (*n* = 4); - C_t_ or Estimated Mean Log SBV Copy Number not defined, as no SBV RNA was detected in the pool.

**Table 2:** The comparison of results of two surveillance approaches: random (23–24 traps randomly distributed over the country [Figure 1]) and targeted (based on five, selected traps). The targeted surveillance between 2013 and 2015 concerns the same five traps as for 2016 and 2017. The results of SBV testing are presented by number of location, number of pools, number of tested individual *Culicoides* and maximum likelihood estimation of infection rate (MLE) with 95% confidence interval (CI).

Year	Random Surveillance				Targeted Surveillance			
	SBV Positive/Tested Location	SBV Positive/Tested Pools	Tested Individuals	MLE (95% CI)	SBV Positive/Tested Location	SBV Positive/Tested Pools	Tested Individuals	MLE (95% CI)
2013	6/23	8/1679	39,586	0.20 (0.09–0.38)	2/5	3/699	16,288	0.18 (0.03–0.50)
2014	9/24	14/1433	34,338	0.40 (0.23–0.67)	1/5	1/475	11,399	0.09 (0.005–0.42)
2015	7/24	7/1460	32,273	0.21 (0.1–0.43)	2/5	2/443	9745	0.21 (0.04–0.67)
2016	-	-	-	-	4/5	35/489	9773	3.7 (2.63–5.09)
2017	-	-	-	-	2/5	2/400	8932	0.22 (0.04–0.73)

**Table 3:** Summary of the overall minimum infection rate (MIR) in *Culicoides* from different studies calculated as the number of positive pools (heads or whole insects) divided by the overall number of tested midges from all tested species, parity statuses, and locations multiplied by 1000.

Period	Country	Positive Pools	Tested Individuals	Overall MIR	Reference
2011	Belgium	38	7305	5.2	[29]
	Germany	0	4999	0.0	[31]
	Netherland	14	6100	2.3	[10]
	Spain	0	5548	0.0	[32]
2011–2012	France	25	29,285	0.9	[32]
	Italy	7	7207	1.0	[11]
	Poland	44	8263	5.3	[8]
2012	Belgium	23	18,820	1.2	[7]
	Germany	2	5562	0.4	[31]
	Netherland	2	6500	0.3	[10]
	Spain	2	1477	1.4	[32]
2013	Germany	0	10,803	0.0	[31]
	Poland	8	39,586	0.2	present study
	Spain	0	566	0.0	[32]
2014	Germany	0	33	0.0	[31]
	Ireland	0	3048	0.0	[21]
	Poland	14	34,338	0.4	present study
2015	Poland	7	32,273	0.2	present study
2016	Belgium	15	2100	7.1	[20]
	Poland	35	9773	3.58	present study
2017	Poland	2	8932	0.2	present study

## Supplementary Materials

The following are available online at https://www.mdpi.com/1999-4915/11/5/447/s1, Figure S1: Standard curve for determination of copy numbers, Table S1: Detailed information on midge abundance.
