# Supplementary material for: Post-Epidemic Distribution of Schmallenberg Virus in Culicoides Arbovirus Vectors in Poland
Source: Viruses. 2019 May 16;11(5):447. doi: 10.3390/v11050447 (PMC6563501; doi:10.3390/v11050447)
Supplement: Supplementary file 1 [file viruses-11-00447-s001.pdf]

viruses

MDPI

Supplementary Materials:

Table S1. Detailed information on midge abundance. Not for all trapped midges parity status was determined. [1]mean midge abundance per trapping site calculated as number of tapped midges in respective category divided by number of trapping sites; [2] mean midge abundance per single night catch calculated as number of tapped midges in respective category divided by number of trapping nights.

Year	Category	Number of Midges Trapped	No of Trapping Sites	Mean Abundance Per Site[1]	No of Night Catches	Mean Abundance Per Catch[2]	%
2013	*C. obsoletus/scoticus* complex	338,254	23	14 706.7	672	503.4	50.8
	C. pulicaris	6751	23	293.5	672	10.0	1.0
	C. punctatus	251,482	23	10 934.0	672	374.2	37.8
	remaining *Culicoides*	69,549	23	3 023.9	672	103.5	10.4
	nulliparous	414,586	23	18 025.5	672	616.9	67.0
	pars	125,103	23	5 439.3	672	186.2	20.2
	blood fed	51,883	23	2 255.8	672	77.2	8.4
	gravid	9681	23	420.9	672	14.4	1.6
	male	17,496	23	760.7	672	26.0	2.8
	total	666,036	23	28 958.1	672	991.1	100.0
2014	*C. obsoletus/scoticus* complex	755,220	24	31 467.5	694	1 088.2	59.8
	C. pulicaris	5964	24	248.5	694	8.6	0.5
	C. punctatus	391,065	24	16 294.4	694	563.5	31.0
	remaining *Culicoides*	111,086	24	4 628.6	694	160.1	8.8
	nulliparous	657,458	24	27 394.1	694	947.3	64.4
	pars	237,329	24	9 888.7	694	342.0	23.2
	blood fed	112,188	24	4 674.5	694	161.7	11.0
	gravid	8097	24	337.4	694	11.7	0.8
	male	6599	24	275.0	694	9.5	0.6
	total	1263,335	24	52 639.0	694	1 820.4	100.0

2015	*C. obsoletus/scoticus* complex	591 620	24	24 650.8	616	960.4	77.3
	C. pulicaris	12 100	24	504.2	616	19.6	1.6
	C. punctatus	78 875	24	3 286.5	616	128.0	10.3
	remaining *Culicoides*	82 588	24	3 441.2	616	134.1	10.8
	nulliparous	411 606	24	17 150.3	616	668.2	59.0
	pars	214 758	24	8 948.3	616	348.6	30.8
	blood fed	56 391	24	2 349.6	616	91.5	8.1
	gravid	9 590	24	399.6	616	15.6	1.4
	male	4 734	24	197.3	616	7.7	0.7
	total	765 183	24	31 882.6	616	1 242.2	100.0
2016	*C. obsoletus/scoticus* complex	194 078	5	8 086.6	163	1 190.7	75.5
	C. pulicaris	835	5	34.8	163	5.1	0.3
	C. punctatus	47 995	5	1 999.8	163	294.4	18.7
	remaining *Culicoides*	13 987	5	582.8	163	85.8	5.4
	nulliparous	148 614	5	29 722.8	163	911.7	60.9
	pars	47 405	5	9 481.0	163	290.8	19.4
	blood fed	42 444	5	8 488.8	163	260.4	17.4
	gravid	3 092	5	618.4	163	19.0	1.3
	male	2 593	5	518.6	163	15.9	1.1
	total	256 895	5	51 379.0	163	1 576.0	100.0
2017	*C. obsoletus/scoticus* complex	274 526	5	11 438.6	142	1 933.3	54.9
	C. pulicaris	8 884	5	370.2	142	62.6	1.8
	C. punctatus	195 112	5	8 129.7	142	1 374.0	39.0
	remaining *Culicoides*	21 665	5	902.7	142	152.6	4.3
	nulliparous	218 235	5	43 647.0	142	1 536.9	43.6
	pars	96 141	5	19 228.2	142	677.0	19.2
	blood fed	167 090	5	33 418.0	142	1 176.7	33.4
	gravid	5 040	5	1 008.0	142	35.5	1.0
	male	13 681	5	2 736.2	142	96.3	2.7
	total	500 187	5	100 037.4	142	3 522.4	100.0

Total 2013–2017	*C. obsoletus/scoticus* complex	2 153 698	81	26 588.9	2 287	941.7	62.4
	C. pulicaris	34 534	81	426.3	2 287	15.1	1.0
	C. punctatus	964 529	81	11 907.8	2 287	421.7	27.9
	remaining *Culicoides*	298 875	81	3 689.8	2 287	130.7	8.7
	nulliparous	1 850 499	81	22 845.7	2 287	809.1	60.0
	pars	720 736	81	8 898.0	2 287	315.1	23.4
	blood fed	429 996	81	5 308.6	2 287	188.0	14.0
	gravid	35 500	81	438.3	2 287	15.5	1.2
	male	45 103	81	556.8	2 287	19.7	1.5
	total	3 451 636	81	42 612.8	2 287	1 509.2	100.0

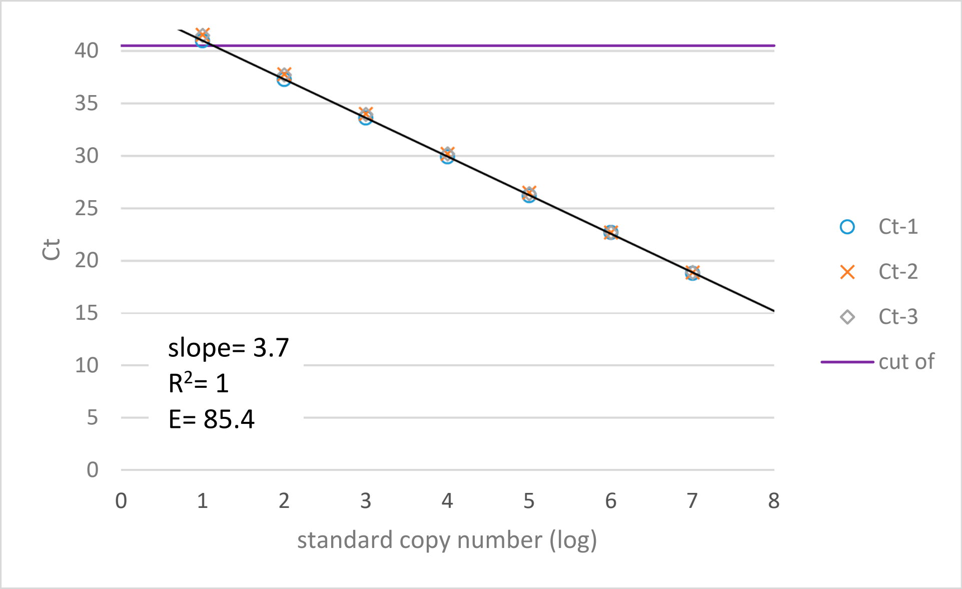

Figure S1. Standard curve for determination of copy numbers. C_t values were plotted against the log of copy number of 87 bp SBV segment S standard obtained using forward TCAGATTGTCATGCCCCTTGC and reverse TTCGGCCCCAGGTGCAAATC starters.
